# Multi-Parameter Collaborative Optimization of Foamed Asphalt Cold Recycling Mixture Properties

**DOI:** 10.3390/ma19102123

**Published:** 2026-05-18

**Authors:** Wei Qiu, Bin Li, Ziyi Song, Xiaoling Zou, Mingjun Hu, Yanqiu Bi

**Affiliations:** 1 Poly Changda Overseas Engineering Co., Ltd., No. 942, Middle of Guangzhou Avenue, Tianhe District, Guangzhou 510000, China; qiuwei@polycdoverseas.com; 2 National & Local Joint Engineering Research Center of Transportation Civil Engineering Materials, Chongqing Jiaotong University, Chongqing 400074, China; bin2020@cqjtu.edu.cn (B.L.); 13592509869@163.com (Z.S.); biyanqiu@chd.edu.cn (Y.B.); 3 School of Civil Engineering, Chongqing Jiaotong University, Chongqing 400074, China; 4 Key Laboratory of Highway Construction & Maintenance Technology in Loess Region, Shanxi Transportation Technology Research and Development Co., Ltd., Taiyuan 030006, China; 5 School of Transportation and Logistics, Dalian University of Technology, Dalian 116024, China; humj0102@163.com

**Keywords:** foamed asphalt, foaming characteristics, cold recycled mixture, indirect tensile strength, influencing factors

## Abstract

This study examined the foaming characteristics of asphalt and their effects on the performance of cold recycled mixtures. The expansion ratio and half-life were used to evaluate effects of asphalt type, foaming temperature, and water content. The influence of asphalt content, gradation, cement content, curing time, and mixing water on mechanical properties and water stability was analyzed. The results indicate that asphalt type is the key factor affecting foaming performance. CNOOA asphalt showed optimal foaming at 160 °C with 2% water, achieving an expansion ratio of 27 and a half-life over 30 s. Optimal asphalt contents for gradations A and B are 3.5% and 2.5%, respectively. A 1.5% cement content provides the best performance balance. Dry and wet indirect tensile strengths increased by 91.18% and 205.56% after 3-day curing. The optimal mixing water ranges are 60–90% and 70–80% of optimum moisture content for gradations A and B. Curing time has the most significant influence on performance, followed by cement and asphalt content. This study provides a theoretical basis for optimizing foamed asphalt cold recycling.

## 1. Introduction

With the continuous development of transportation systems, many asphalt pavements constructed in earlier periods have gradually entered the maintenance stage. The rehabilitation and reconstruction of aged asphalt pavements generate more than 200 million tons of reclaimed asphalt pavement (RAP) materials annually [1,2]. The large-scale stockpiling of RAP not only occupies limited land resources but also causes direct pollution and damage to the ecological environment [3]. Therefore, the recycling and reuse of RAP has become a promising approach for conserving virgin resources and reducing pavement construction and maintenance costs, and it has increasingly become a key research focus among scholars in the field of road engineering.

RAP recycling technologies can be classified into two categories according to the recycling process and mixing temperature [4,5,6]: hot recycling and cold recycling technologies [7]. Compared with hot recycling, cold recycling exhibits more pronounced economic and environmental benefits [7]. Economically, this technology enables the effective reuse of more than 70% of RAP and achieves high recovery rates of both coarse and fine aggregates [8]. Environmentally, because mixing, paving, and compaction are conducted at relatively low temperatures, cold recycling plays an important role in reducing carbon emissions during highway infrastructure construction [9]. According to the type of stabilizing agent used, cold recycling technology can be further divided into emulsified asphalt cold recycling and foamed asphalt cold recycling [10,11]. Compared with emulsified asphalt recycling, foamed asphalt cold recycling shows clear advantages in pavement performance and construction efficiency. In terms of performance, foamed asphalt can effectively adhere to the surface of RAP aggregates and form a high-strength flexible skeleton, thereby endowing the recycled mixture with higher compressive strength and deformation resistance [12]. In terms of construction efficiency, foamed asphalt mixtures exhibit good mixing uniformity, provide a longer workable compaction time after paving, and eliminate the need for a prolonged demulsification period, thereby significantly shortening the curing period before opening to traffic [13]. These prominent advantages have jointly promoted the continuous expansion of engineering applications of foamed asphalt cold recycling technology [12,14]. Compared with conventional hot-mix asphalt mixtures, foamed asphalt-stabilized materials differ substantially in composition structure, asphalt dispersion form, moisture state, and strength formation mechanism. Therefore, their mix design cannot directly adopt the conventional design framework for hot-mix asphalt mixtures; instead, factors such as gradation, moisture content, active filler, and curing process must be comprehensively considered [15,16,17]. However, foamed asphalt cold reclaimed mixtures (FACRM) still face several challenges that need to be addressed, including low early strength, prolonged curing time, and insufficient moisture resistance [13,18].

To improve the strength of FACRM, extensive studies have been conducted on asphalt foaming characteristics as well as the material composition and construction control parameters of FACRM. In terms of foaming characteristics, the foaming behavior of asphalt is directly related to the workability and performance of recycled mixtures [19]. Foamed asphalt mixtures themselves exhibit pronounced temperature dependence and temperature sensitivity, and the quality of asphalt foaming performance is a key factor governing the resistance of such mixtures to temperature variation and moisture-induced damage [20,21]. Moreover, asphalts from different sources and with different penetration grades exhibit inherent differences in foaming characteristics. Variations in foaming capacity can directly lead to marked differences in the temperature sensitivity and moisture susceptibility of recycled mixtures [22]. In addition, asphalt foaming characteristics are closely related to the foaming water content. Specifically, at a given asphalt temperature, the expansion ratio of foamed asphalt generally increases with increasing foaming water content, whereas the half-life decreases accordingly [23]. Regarding the material composition and construction control parameters of FACRM, gradation characteristics play a crucial role in determining the mechanical properties of the mixture. As the sieve size decreases, the influence of the passing percentage becomes more pronounced. It has been reported that increasing the content of fine fractions can effectively improve the mechanical performance of the mixture [10,24]. Active fillers also play a key role in FACRM. The hydration reaction of cement can substantially enhance the strength, resilient modulus, and resistance to moisture damage of the mixture. However, an excessive cement content may excessively increase the stiffness of the mixture, thereby weakening its resistance to temperature-induced shrinkage cracking. Therefore, to balance stiffness and cracking resistance, the cement content is generally recommended to be controlled within 2% [25,26]. Physical binders also affect the performance of FACRM. After foaming, the temporary reduction in asphalt viscosity and the significant increase in surface area facilitate sufficient coating and penetration of RAP aggregates [27]. Moreover, the strength of FACRM is closely associated with the mixing water content, and it increases as moisture evaporates during curing [28]. Curing is another critical stage that determines the performance of foamed asphalt cold recycled pavements, as it provides the necessary time for the development of asphalt bonding, the progression of cement hydration, and the evaporation of excess moisture. An adequate curing period is essential; in practice, the completion of curing is commonly judged by whether the moisture content of the mixture has decreased to a specified threshold [12]. Although extensive studies have been conducted on the pavement performance of FACRM, the understanding of its comprehensive damage evolution process remains incomplete. In addition, the explicit relationship between internal structural damage and mechanical damage in cold recycled mixtures requires further investigation. Although foamed asphalt cold recycled mixtures have been widely studied, several limitations remain in the existing literature [29,30]. First, previous studies have mostly examined the effects of asphalt type, foaming temperature, foaming water content, gradation, cement content, or curing conditions on foaming performance or mixture performance from a single-factor perspective, while insufficient attention has been paid to the continuous interaction between asphalt foaming characteristics and the strength and moisture stability of recycled mixtures. Second, different material parameters and construction control parameters often jointly affect the performance of foamed asphalt cold recycled mixtures; however, quantitative comparisons of the relative contribution of each factor remain limited. As a result, mix design optimization and construction parameter control still rely, to some extent, on empirical judgment. In particular, under different asphalt sources and gradation conditions, how to further coordinate asphalt content, mixing water content, cement content, and curing time while satisfying the requirements for foaming performance still requires more systematic experimental verification and statistical analysis.

**Figure 1:**
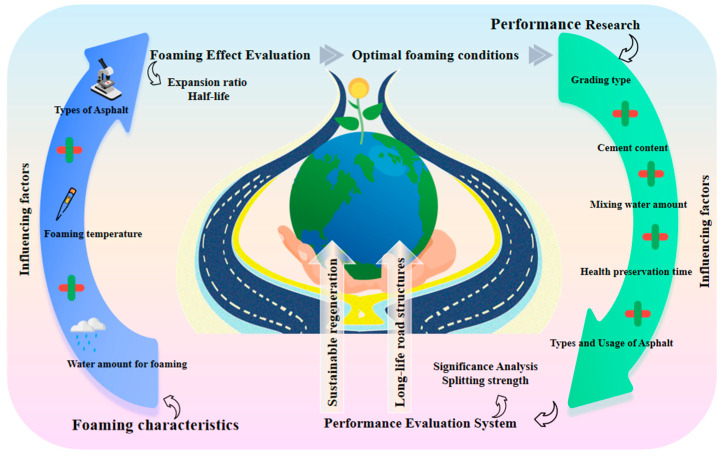
Technical Flow Chart.

Based on the aforementioned issues, this study focuses on foamed asphalt cold recycled mixtures. First, using the expansion ratio and half-life as evaluation indices, the effects of asphalt type, foaming temperature, and foaming water content on asphalt foaming performance were systematically investigated, and the recommended foaming conditions for subsequent mixture tests were determined. On this basis, a multifactor experimental program was further designed, incorporating asphalt type and content, gradation type, mixing water content, curing time, and cement content. The mechanical properties and moisture stability of the recycled mixtures were evaluated using dry and wet indirect tensile strength and the tensile strength ratio. Unlike studies that merely compare mixture performance based on single-factor analysis, this study further introduces Pearson correlation analysis, one-way analysis of variance, and multiple linear regression to quantitatively identify the contribution of key factors to strength development and to establish statistical relationships between key parameters and indirect tensile strength. The focus of this study is not to propose a new cold recycling material system, but rather to reveal the synergistic effects of multiple parameters on the performance of foamed asphalt cold recycled mixtures through a unified experimental framework and statistical analysis methods. The findings are expected to provide a clearer basis for material selection, mix design, and construction parameter control. The technical flowchart is shown in Figure 1.

## 2. Materials and Methods

### 2.1. Materials

#### 2.1.1. Foamed Asphalt

In this study, asphalt foaming tests were conducted using three types of asphalt: Korean SK 70# asphalt (KA), CNOOC 70# Grade A asphalt (CNOOA), and Donghai 70# Grade A asphalt (ECSA). Their key technical properties were determined in accordance with the Standard Test Methods of Asphalt and Bituminous Mixtures for Highway Engineering (JTG E20-2011) [31], and the results are summarized in Table 1.

#### 2.1.2. Recycled Mixture and Gradation

**Figure 2:**
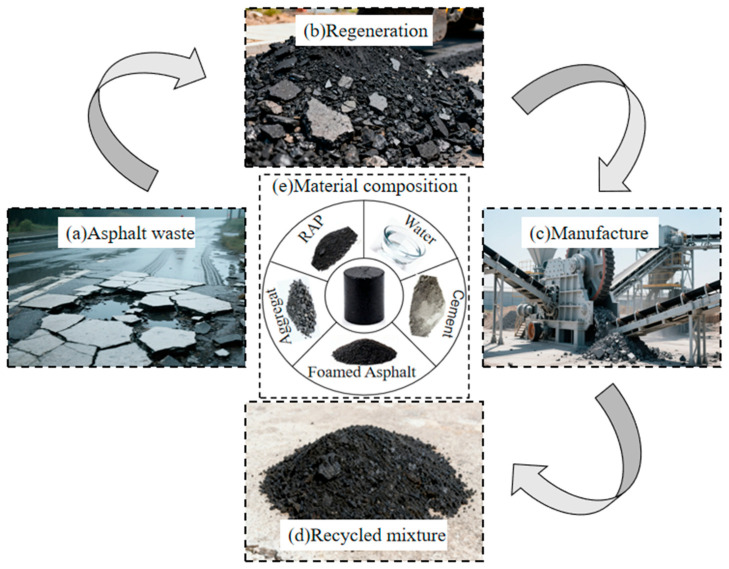
RAP Acquisition Flow Chart.

**Figure 3:**
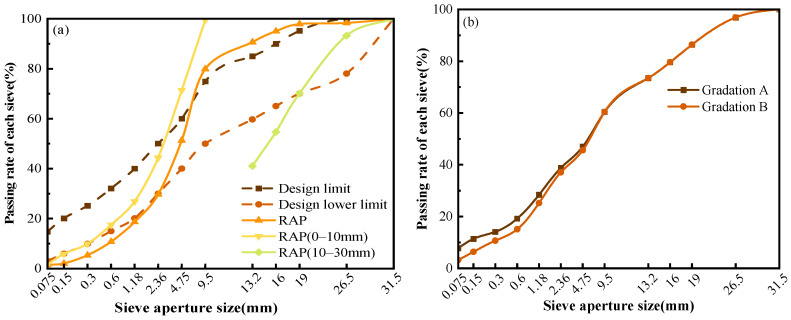
Particle Size Distribution Curves: (**a**) RAP; (**b**) Gradation Curve.

The RAP used in this study was obtained from a major and medium rehabilitation project of an expressway in Chongqing through cold milling, as shown in Figure 2. Its key properties, including moisture content, water absorption, Los Angeles abrasion loss, apparent relative density, and bulk specific gravity, were 1.8%, 1.3%, 22.8%, 2.681, and 2.594, respectively. The gradation results of the RAP are presented in Figure 3a.To meet the gradation requirements for granular FACRM specified in the Technical Specifications for Highway Asphalt Pavement Recycling (JTG F41-2008 [33]), two synthetic gradation schemes, denoted as gradations A and B, were designed in this study, as shown in Table 2 and Figure 3b. Gradation A incorporated 20% mineral filler, whereas no mineral filler was added to gradation B. In this paper, the term “mineral filler” refers to fine mineral powder passing the 0.075 mm sieve, which is mainly used to increase the fine-particle content in foamed asphalt cold recycled mixtures, fill the voids between aggregates, and promote the dispersion and bonding of foamed asphalt on the surface of fine aggregates. To control variables, the gradation design was intended to minimize interference from factors other than the passing percentage of filler particles. According to the specification, the cement content should generally not exceed 2%. Therefore, the cement content used in this study was determined as 1.5% of the total mass of the mineral aggregate mixture, excluding cement. China Chongqing Xiaqiang P.C 32.5 composite Portland cement was used in this experiment. Selected standard tests were conducted on the cement, and the results are listed in Table 3.

#### 2.1.3. Optimum Moisture Content

The optimal moisture content was determined via the heavy compaction test, while the optimal foamed asphalt content was determined based on the peak value of wet indirect tensile strength. The corresponding results are summarized in Table 4.

#### 2.1.4. Preparation of Foamed Asphalt and FACRM

**Figure 4:**
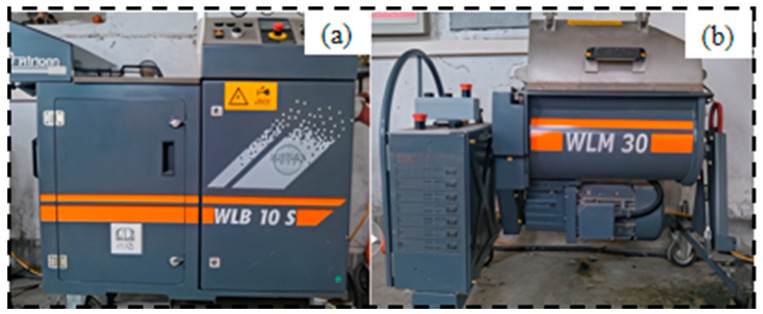
Wirtgen Asphalt Foaming Device and Mixing Machine: (**a**) Foaming Equipment; (**b**) Mixing Machine.

The preparation of foamed asphalt was performed using specialized foaming equipment, and the mixing of cold recycled mixtures was carried out using corresponding supporting devices. In this experiment, the German Wirtgen WLB10S laboratory foaming equipment and WLM30 mixer were used for relevant operations, and the experimental devices are shown in Figure 4.

**Figure 5:**
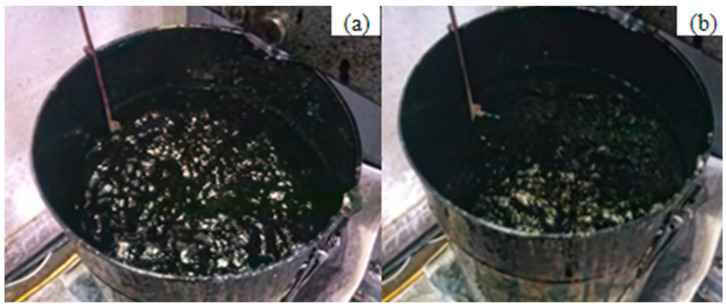
Schematic Diagram of Foaming Effect: (**a**) State of Maximum Expansion Ratio; (**b**) State of Half-Life.

The foamed asphalt test was conducted as follows. First, clean tap water at room temperature was injected, and a stable air supply was connected. The test air pressure, water pressure, and foamed asphalt discharge rate were set to 4 bar, 5 bar, and 100 g/s, respectively. Subsequently, the asphalt tank was preheated and filled with asphalt. When the asphalt temperature reached 150 °C, the circulation pump was started, and heating was continued for 5 min. The asphalt flow rate was then calibrated at a discharge rate of 100 g/s with a discharge mass of 500 g, and the foaming water content was determined accordingly. Finally, the formal foaming test was performed. The foamed asphalt was sprayed into a test bucket, and the expansion ratio was recorded when the foam volume reached its maximum. At the same time, a stopwatch was started, and the time required for the foam volume to decrease to half of its maximum value was recorded as the half-life. Each test was repeated three times, and the average values of the expansion ratio and half-life were used to evaluate the foaming performance. A schematic diagram of the foaming effect is shown in Figure 5.

The preparation procedure of FACRM was as follows. First, the reclaimed materials and coarse RAP fraction of 10–30 mm were placed into the mixing tank. Then, approximately 1/5–1/4 of the total mixing water and 1/3 of the foamed asphalt were added, and the mixture was blended for 60 s. Subsequently, the remaining water was used to moisten the fine RAP fraction of 0–10 mm by manual mixing. The pre-wetted fine RAP and the remaining foamed asphalt were then added to the mixing tank and mixed for 136 s. Finally, cement and mineral filler were simultaneously introduced into the mixing tank and mixed for another 60 s, yielding the FACRM. After the above mixing process, the prepared mixture was compacted into standard Marshall specimens with a diameter of 63.5 mm and a height of 101.6 mm.

### 2.2. Methods

#### 2.2.1. Asphalt Foaming Test Scheme

To investigate the effects of asphalt temperature and foaming water content on the foaming performance of different asphalts, a series of experiments was conducted using a Wirtgen WLB10S foaming device on three types of asphalt, namely KA, CNOOA, and ECSA. The foaming performance under various conditions was evaluated based on specific performance indicators, with the aim of determining the optimal foaming conditions. In this study, three asphalts (KA, CNOOA, and ECSA) were tested at foaming temperatures of 150 °C, 160 °C, and 170 °C, combined with different foaming water contents (1%, 2%, 3%, and 4%). The detailed experimental design is presented in Table 5.

#### 2.2.2. Effect Evaluation of Asphalt Foaming

**Figure 6:**
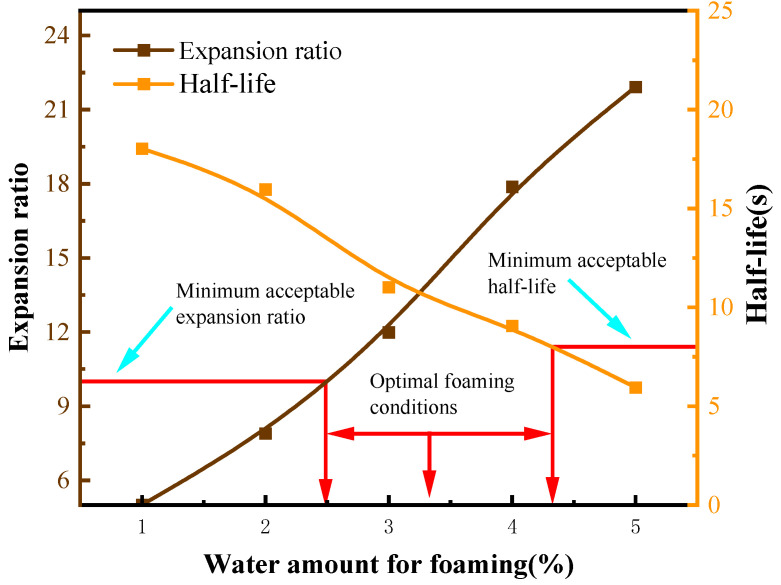
Determination Method of Optimum Asphalt Foaming Conditions.

Based on the mechanism of asphalt foaming and collapse, expansion ratio and half-life were adopted as the core indicators for evaluating foaming performance in this study [17]. The laboratory foaming tests were conducted to determine the optimal combination of process parameters that can simultaneously achieve a large expansion ratio and a long half-life, among which asphalt temperature and foaming water content are regarded as two key control factors. The optimal foaming conditions refer to the parameter combination corresponding to the asphalt’s optimal foaming effect (i.e., the comprehensive optimization of expansion ratio and half-life). In this study, the specific optimal conditions were determined by referring to the relevant domestic and foreign literature [38] and the test results shown in Figure 6. Among them, the ideal foaming effect standard recommended in the Wirtgen Cold Recycling Manual [39] is an expansion ratio not less than 10 and a half-life no less than 8 s.

As shown in Figure 6, at a given temperature, the relationship between asphalt foaming characteristics (expansion ratio and half-life) and foaming water content can be described by inverse functions, and the corresponding curves have been plotted. The determination of the optimal foaming condition for foamed asphalt should consider both the expansion ratio and the half-life. The expansion ratio reflects the volumetric expansion capacity of asphalt after foaming and its potential for dispersion within recycled mixtures, while the half-life represents the stability of the foam structure and the available mixing time. According to the foamed asphalt design method proposed by Jenkins [40], as well as the TG2 and Wirtgen cold recycling guidelines [39], the optimum foaming water content should not be determined based on a single indicator, nor simply regarded as the intersection point of the expansion ratio and half-life curves. Instead, it should be selected comprehensively on the premise that both the minimum requirements for expansion ratio and half-life are satisfied [41].

#### 2.2.3. Indirect Tensile Test Method

**Figure 7:**
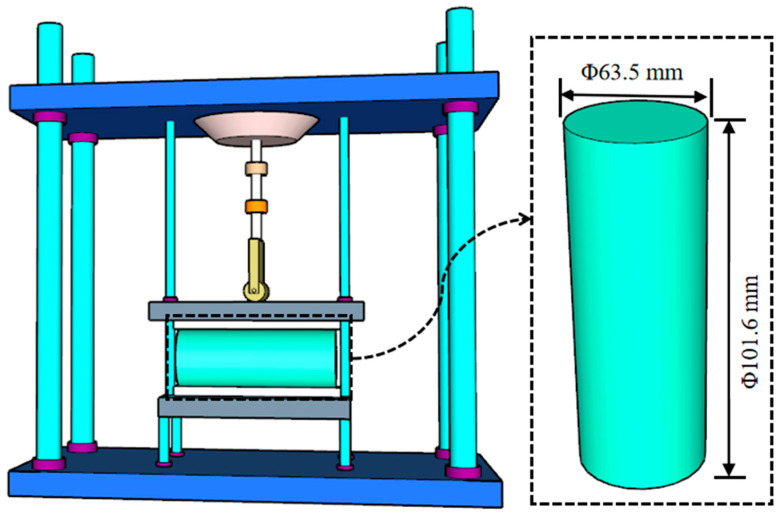
Loading Setup of dry and wet indirect tensile strength Test.

To evaluate the moisture stability of foamed asphalt cold recycled mixtures, the indirect tensile strength (ITS) test under dry and wet conditions was conducted in accordance with the Standard Test Methods of Asphalt and Bituminous Mixtures for Highway Engineering (JTG E20-2011) [31]. First, cylindrical specimens were prepared using the Marshall compaction method. At room temperature, the Marshall specimens, together with the molds, were left to cure for 24 h. After demolding, the specimens were placed in a ventilated oven at 40 °C for 72 h of constant-temperature conditioning. The conditioned specimens were then divided into two groups: a dry group and a wet group. The dry specimens were stored in an oven at 5 ± 0.5 °C until testing. Wet specimens were immersed in a water bath at 25 ± 0.5 °C for 24 h, followed by conditioning in an oven for an additional 2 h. Three parallel specimens were prepared and tested for each group, and the final results were obtained as the average of the three measurements. Subsequently, the specimens were tested using a universal testing machine equipped with an indirect tensile loading fixture. The tests were conducted at 25 °C with a loading rate of 50mm/min until failure, and the peak load was recorded. Finally, the indirect tensile strength under dry and wet conditions was calculated based on the test results, and the indirect tensile strength ratio (ITSR) was used as the final indicator to evaluate moisture stability. The loading configuration for the indirect tensile test is illustrated in Figure 7.

#### 2.2.4. Significance Analysis Method

To improve the reliability of the experimental analysis, statistical analysis was further conducted in this study. Three parallel specimens were prepared for each test group, and the results are presented as the mean ± standard deviation. The error bars in the figures represent the standard deviation of the three repeated measurements. With indirect tensile strength as the dependent variable and asphalt content, curing time, and cement content as independent variables, Pearson correlation analysis was first employed to evaluate the relationships between these factors and the indirect tensile strength. One-way analysis of variance (ANOVA) was then conducted to determine whether the differences among the test groups were statistically significant, with the significance level set at *p* < 0.05. When *p* < 0.05, the differences between groups were considered statistically significant. On this basis, multiple linear regression analysis was further performed to quantify the influence of each factor on the indirect tensile strength and to establish a predictive model. All statistical analyses were carried out using IBM SPSS Statistics 26 software.

## 3. Results and Discussion

All curves presented in this study are derived from the averaged experimental data points under each test condition and plotted using a spline fitting method to illustrate the trends of the performance indicators under different influencing factors. Only in 3.1.4. Determination of Optimum Asphalt Foaming Conditions is an inverse function model employed to fit the variations in the expansion ratio and half-life as functions of the foaming water content.

### 3.1. Analysis of Asphalt Foaming Test Results

#### 3.1.1. Influence of Asphalt Types on Foaming Performance

**Figure 8:**
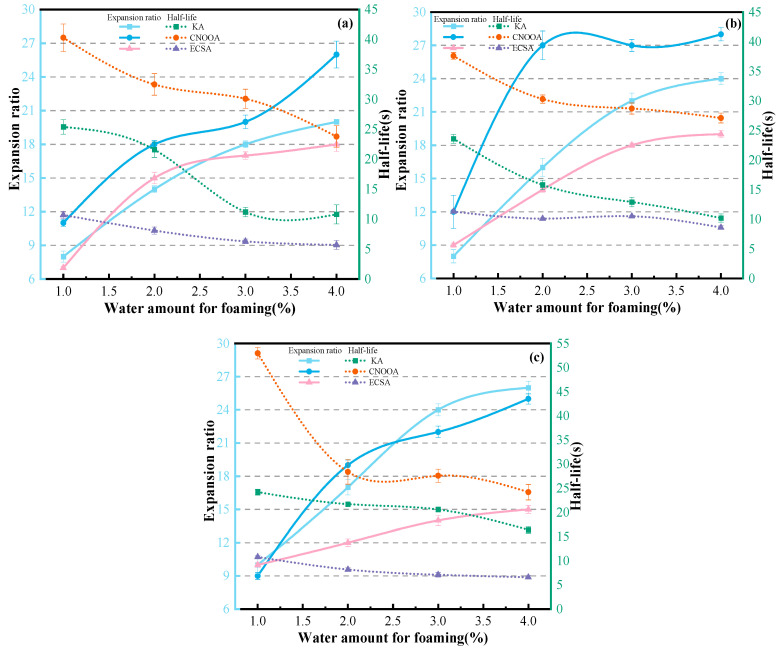
Relationship Between Asphalt Types, Expansion Ratio and Half-Life Under Different Asphalt Temperatures: (**a**) 150 °C; (**b**) 160 °C; (**c**) 170 °C.

Figure 8 shows the relationship curves between the expansion ratio, half-life of different asphalt types and foaming water content under different asphalt temperatures.

As shown in Figure 8a, at an asphalt temperature of 150 °C, when the foaming water content is 1% and 3%, CNOOA exhibits the highest expansion ratio, followed by KA, while ECSA shows the lowest performance. When the foaming water content is 2% and 4%, CNOOA still presents the highest expansion ratio, followed by ECSA, with KA being the lowest. Notably, at a foaming water content of 4%, compared with CNOOA, the expansion ratios of KA and ECSA decrease by 23.08% and 30.77%, respectively, representing the largest difference. In contrast, at a foaming water content of 3%, the reductions are 10.00% and 15.00%, respectively, corresponding to the smallest difference. In terms of half-life, CNOOA consistently shows the highest values, followed by KA, while ECSA performs the worst. When the foaming water content is 1%, all three asphalts exhibit their maximum half-life values, reaching 40.3 s, 23.6 s, and 10.7 s, respectively. Similarly, as shown in Figure 8b,c, at an asphalt temperature of 160 °C, when the foaming water content is greater than or equal to 2%, CNOOA demonstrates the highest expansion ratio, followed by KA, with ECSA being the lowest. When the foaming water content is 1%, compared with CNOOA, the expansion ratios of KA and ECSA decrease by 33.33% and 25.00%, respectively, representing the smallest difference. Regarding half-life, CNOOA again shows the highest values, followed by KA and then ECSA. At a foaming water content of 1%, the half-life values of the three asphalts reach their maximums, which are 37.6 s, 23.6 s, and 11.3 s, respectively. At an asphalt temperature of 170 °C, when the foaming water content is 1%, the expansion ratios of KA and ECSA are equal at 10, while that of CNOOA is 8. When the foaming water content is 3% and 4%, KA exhibits the highest expansion ratio, followed by CNOOA, with ECSA remaining the lowest. However, in terms of half-life, CNOOA still shows the highest values, followed by KA and then ECSA. Overall, at 150 °C, KA and ECSA exhibit similar expansion ratios, but KA has a longer half-life. At 160 °C, CNOOA shows significantly higher expansion ratio and half-life than both KA and ECSA, with particularly superior performance in half-life. At 170 °C, although the difference in expansion ratio between KA and CNOOA is relatively small, the half-life of KA is notably lower than that of CNOOA. Meanwhile, both KA and CNOOA outperform ECSA in terms of both expansion ratio and half-life.

According to the recommendations in the Wirtgen Cold Recycling Manual (2004), the reference criteria for expansion ratio and half-life are 10 and 8 s, respectively, which can serve as basic benchmarks for evaluating whether the foaming performance is acceptable. Within the foaming water content range of 1–4%, KA exhibits favorable foaming performance at temperatures of 160–170 °C with water contents of 3–4%; CNOOA shows significant foaming performance at temperatures of 150–160 °C with water contents of 2–3%; similarly, ECSA also demonstrates good foaming performance at temperatures of 150–160 °C with water contents of 2–3%. The experimental results indicate that different types of asphalt exhibit significant differences in foaming performance under the same foaming conditions. These differences are primarily attributed to variations in the chemical composition of the asphalts, particularly the physical properties strongly influenced by these components [30]. The type and origin of crude oil are key factors leading to differences in asphalt composition, which in turn affect foaming performance [42]. Even for the same type of asphalt, when conventional technical indices are similar, but the source differs, significant variations in foaming performance may still occur [43].

#### 3.1.2. Influence of Asphalt Temperature on Foaming Performance

**Figure 9:**
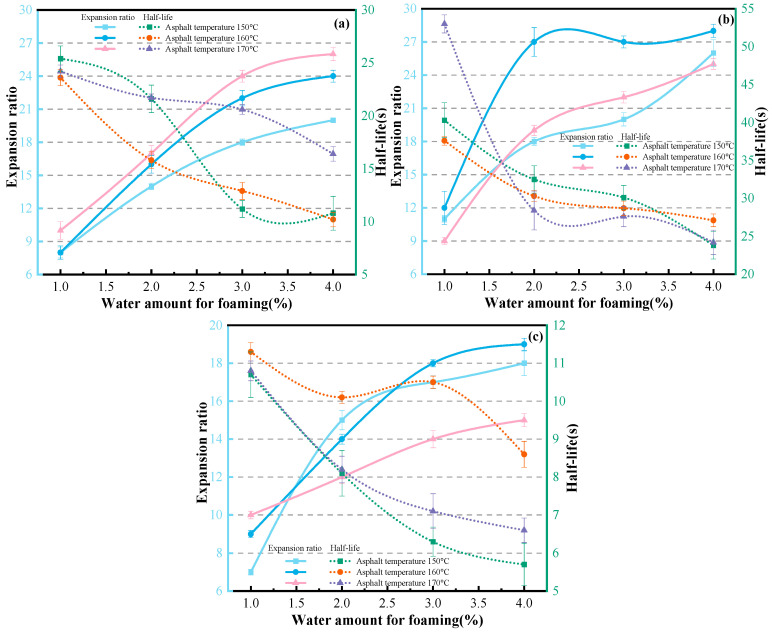
Influence of Asphalt Temperature on Foaming Indicators Under Different Asphalt Types: (**a**) KA; (**b**) CNOOA; (**c**) ECSA.

Figure 9 shows the relationship curves between the foaming indicators of KA, CNOOA, and ECSA and foaming water content under different asphalt temperatures.

As shown in Figure 9a, with increasing temperature, the expansion ratio of KA exhibits an overall increasing trend at the same foaming water content. When the foaming water content is 1%, the expansion ratios at 150 °C and 160 °C are identical, both equal to 8. At a foaming water content of 3%, compared with 150 °C, the expansion ratio at 160 °C shows the largest increase, reaching 22.22%. When the foaming water content is 4%, the expansion ratios of KA increase to 20, 24, and 26 with rising temperature, reaching the maximum values. In terms of half-life, under the same foaming water content, no clear trend is observed with increasing temperature. When the foaming water content is 1%, the effect of temperature on half-life is minimal, with values of 25.4 s, 23.6 s, and 24.2 s, respectively. When the foaming water content is 3%, the influence of temperature on half-life is most pronounced, with values of 11.2 s, 12.9 s, and 20.6 s, respectively. Similarly, as shown in Figure 9b, the expansion ratio of CNOOA initially increases and then decreases with increasing temperature at the same foaming water content. When the foaming water content is 2%, the variation in expansion ratio with temperature is the most significant, with values of 18, 27, and 19, respectively. When the foaming water content is 1% and 4%, the variation in expansion ratio with temperature is minimal. Regarding half-life, when the foaming water content exceeds 2%, the influence of temperature on half-life is not significant. As shown in Figure 9c, when the foaming water content exceeds 1.5%, the expansion ratios of ECSA at 150 °C and 160 °C show little difference, but are both higher than that at 170 °C. When the foaming water content is 3%, the influence of temperature on the expansion ratio of ECSA is the most significant, with values of 17, 18, and 14, respectively. For half-life, under the same foaming water content, the half-life of ECSA shows a trend of first increasing and then decreasing with increasing temperature. When the foaming water content is 3%, the influence of temperature on half-life is the greatest, increasing from 6.3 s to 10.5 s and then decreasing to 7.1 s.

In summary, for KA, under the same foaming water content, the expansion ratio of foamed asphalt increases with increasing asphalt temperature [44]. When the foaming water content is 3%, the half-life shows significant variation. At an asphalt temperature of 170 °C, both the expansion ratio and half-life of KA are markedly superior to those at other temperatures. For CNOOA, at the same foaming water content, the expansion ratio at 160 °C is significantly higher than that at 150 °C and 170 °C. When the foaming water content exceeds 2%, the effect of asphalt temperature on half-life is not significant. For ECSA, when the foaming water content exceeds 1.5%, the expansion ratios at 150 °C and 160 °C show little difference, but are both higher than that at 170 °C. The half-life at 160 °C is significantly higher than that at 150 °C and 170 °C. When the foaming water content exceeds 2%, the influence of asphalt temperature on half-life becomes more pronounced.

The three asphalts, KA, CNOOA, and ECSA, exhibit distinct foaming characteristics at different foaming temperatures, which is mainly attributed to their respective optimal foaming temperature ranges [14]. When the temperature is below this range, the water in the foaming chamber cannot be fully vaporized, making it difficult to generate a sufficient number of vapor bubbles, thereby resulting in poorly developed foam structures. Conversely, when the temperature is excessively high, although foam generation is promoted, bubbles with excessively large diameters are formed. This leads to a reduction in the apparent viscosity of the asphalt and a decrease in the elasticity of the asphalt film, thereby accelerating foam collapse, which is manifested as a significant reduction in half-life and ultimately deteriorates the foaming quality [45].

#### 3.1.3. Influence of Foaming Water Content on Foaming Performance

Figure 9 also illustrates the relationship between foaming indices and asphalt temperature for KA, CNOOA, and ECSA under different foaming water contents.

As shown in Figure 9a, for KA at an asphalt temperature of 170 °C, the expansion ratio increases significantly as the foaming water content rises from 1% to 3%, with increments of 70% and 41.2%, respectively. Beyond 3%, the rate of increase becomes moderate, at only 8.3%. Meanwhile, the half-life remains relatively stable, indicating that KA is better suited for operation at relatively higher water contents of 3–4%. Similarly, as illustrated in Figure 9b, for CNOOA at an asphalt temperature of 160 °C, when the foaming water content increases from 2% to 4%, the expansion ratio remains nearly constant, while the half-life shows only slight reductions of 5.3% and 5.8%, respectively. This suggests that its foaming performance is less sensitive to variations in water content, with an optimal water content range of 2–3%. As shown in Figure 9c, for ECSA at an asphalt temperature of 160 °C, the increase in expansion ratio gradually diminishes with increasing water content, with increments of 55.6%, 28.6%, and 5.5%, respectively. The half-life first decreases and then increases within the water content range of 2–4%, with relatively small overall fluctuations, indicating good controllability of the foaming process.

In summary, with increasing foaming water content, the expansion ratios of all three asphalts exhibit an increasing trend, while the half-life generally decreases, indicating a clear inverse relationship between the two [46]. This also suggests that water, acting as a foaming agent, rapidly vaporizes at high temperatures and serves as the primary driving force for asphalt volume expansion. However, an increase in its content also reduces the stability of the bubble structure, thereby accelerating foam decay [21].

#### 3.1.4. Determination of Optimum Asphalt Foaming Conditions

**Figure 10:**
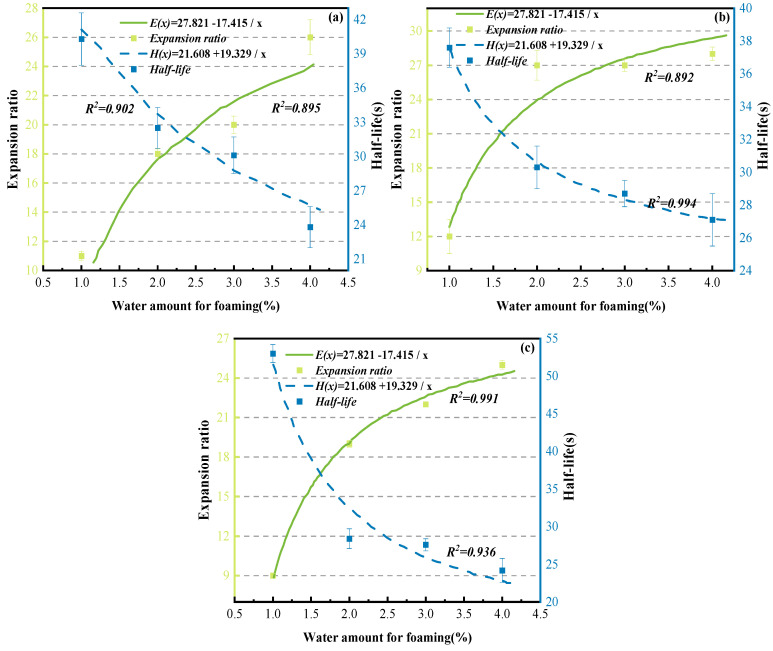
Foaming Characteristic Curves of CNOOA Under Different Asphalt Temperatures: (**a**) 150 °C; (**b**) 160 °C; (**c**) 170 °C.

To identify the key parameters in the asphalt foaming process, this study systematically investigated the effects of asphalt type, foaming temperature, and foaming water content on foaming performance. A comprehensive comparison indicates that CNOOA exhibits the best foaming performance, followed by KA, while ECSA performs relatively poorly. Therefore, CNOOA was selected as the binder for recycled mixtures, and its optimal foaming conditions were further analyzed. To ensure the comparability of fitting results under different temperature conditions, an inverse function model was uniformly adopted to fit the relationships between expansion ratio and half-life with respect to foaming water content. In this study, E(x) represents the fitted function of the expansion ratio, H(x) represents the fitted function of the half-life, and x denotes the foaming water content. The inverse function model was selected because, with increasing foaming water content, the number of vapor bubbles generated by water vaporization increases, leading to a gradual increase in the expansion ratio, while the rate of increase progressively diminishes. Meanwhile, excessive water weakens the stability of the foam structure, resulting in a decrease in half-life that gradually approaches a stable value. Therefore, the inverse function model can reasonably characterize the nonlinear relationship between the enhancement of expansion capability and the attenuation of foam stability during the foaming process of asphalt. The foaming characteristic curves under different temperature conditions are shown in Figure 10.

With increasing temperature, the viscosity of asphalt decreases, allowing foaming water to more easily penetrate into the asphalt and rapidly vaporize [47], which, within a certain range, is beneficial for improving both the expansion ratio and half-life. This study shows that when the temperature increases from 150 °C to 160 °C, the viscosity of CNOOA decreases, facilitating uniform water dispersion and vapor expansion, thereby resulting in superior overall foaming performance near the optimal foaming water content. However, when the temperature further increases to 170 °C, the foaming performance deteriorates [14]. This can be attributed to several factors. On the one hand, excessively low viscosity weakens the ability of the asphalt film to envelop and support the bubbles, leading to unstable foam structures, where bubbles are more prone to coalescence, rupture, and collapse, resulting in a significant reduction in half-life [48]. On the other hand, water vaporizes too rapidly at high temperatures, causing steam to escape quickly, making the foaming process overly intense and difficult to maintain a stable structure [49]. In addition, high temperatures may accelerate the volatilization of light components and short-term thermal aging, altering the colloidal structure of asphalt and further reducing foam stability. Therefore, the foaming performance of CNOOA does not increase monotonically with temperature, but instead shows a trend of first increasing and then decreasing, indicating the existence of an optimal temperature range. Considering both expansion ratio and half-life, 160 °C is identified as the optimal foaming temperature. In determining the optimal foaming conditions, it is necessary to simultaneously consider expansion ratio, half-life, and practical moisture control requirements. Under the conditions of 150 °C/3% and 160 °C/2%, the half-life values are approximately 30.1 s and 30.3 s, respectively, showing minimal difference and both satisfying foam stability requirements. However, the expansion ratio at 160 °C/2% reaches 27, which is significantly higher than the value of 20 at 150 °C/3%, indicating stronger volumetric expansion capacity and dispersion potential. Meanwhile, the lower foaming water content at 160 °C/2% is advantageous in reducing excess moisture in the mixture and lowering subsequent drainage requirements during curing. Excessive water content may lead to unstable foam structures, accelerate bubble coalescence and rupture, and consequently reduce foaming performance. The optimal foaming water content should lie within a range that balances both expansion capacity and stability [50]. Based on the above analysis, by selecting parameter combinations that meet the minimum requirements for expansion ratio and half-life and comparing the overall foaming performance under different temperature and water content conditions, it is concluded that CNOOA exhibits the best comprehensive foaming performance at 160 °C with a foaming water content of 2%. This condition is therefore recommended for subsequent experiments on foamed asphalt cold recycled mixtures.

### 3.2. Analysis of Influencing Factors on Performance of Cold-Recycled Bituminous Mixture

Based on the optimal foaming conditions determined above, further experiments were designed to investigate the factors influencing the performance of foamed asphalt cold recycled mixtures (FACRM). Under two gradation conditions (A and B), the effects of asphalt type and content, mixing water content, curing time, and cement content were systematically examined to analyze their relationships with the performance of recycled mixtures. Specifically, two types of asphalt, CNOOA and ECSA, were used. Different levels of asphalt content, mixing water content, curing time, and cement content were set, and the main parameters and their ranges are presented in Table 6. Among them, CNOOA was used for a systematic analysis of the effects of various factors on FACRM performance, while ECSA was primarily employed to investigate the influence of asphalt content on dry and wet indirect tensile strength under different gradations. For ECSA, the asphalt contents were set at 2.0%, 2.5%, 3.0%, 3.5%, and 4.0% under gradation A, and 1.0%, 1.5%, 2.0%, 2.5%, and 3.0% under gradation B. It should be noted that the mixing water content percentage described in this study does not represent the absolute moisture content, but rather a proportion relative to the optimum moisture content (OMC) determined by the heavy compaction test. The OMC values for gradations A and B are 6.32% and 5.60%, respectively. Therefore, the values of 30%, 50%, 60%, 70%, 80%, 90%, 110%, and 130% used in the experiments represent corresponding proportions of the OMC. After conversion to actual mixing moisture content, the ranges are 1.90–8.22% for gradation A and 1.68–7.28% for gradation B. Among these, 70% OMC corresponds to actual mixing water contents of 4.42% and 3.92% for gradations A and B, respectively.

#### 3.2.1. Influence of Asphalt Types and Dosage on Mixture Performance

**Figure 11:**
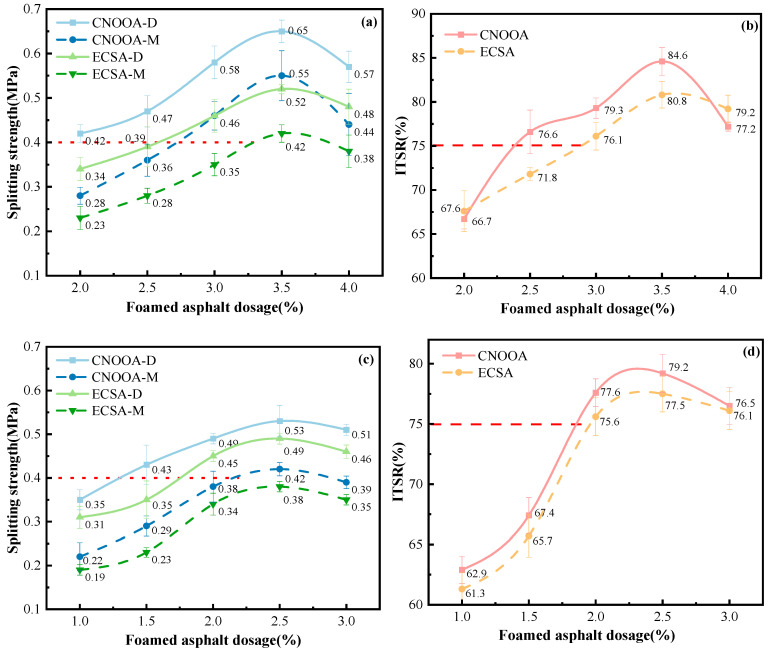
Influence of Asphalt Types and Foamed Asphalt Content on Indirect Tensile Strength Under Different Gradations: (**a**,**b**) Gradation A; (**c**,**d**) Gradation B.

Figure 11 illustrates the effects of asphalt type and asphalt content on the dry and wet indirect tensile strength of FACRM under different gradations. In the figure, “XXX-D” denotes the dry indirect tensile strength, while “XXX-M” represents the wet indirect tensile strength. According to the relevant requirements specified in the Technical Specification for Recycling of Asphalt Pavement, red dashed lines are plotted to indicate the design criteria: at 15 °C, the indirect tensile strength should not be less than 0.4 MPa, and the indirect tensile strength ratio (ITSR) should not be less than 75%. These criteria are adopted as the design control standards for foamed asphalt cold recycled mixtures to ensure adequate strength performance.

According to the test results shown in Figure 11, the indirect tensile strength characteristics of CNOOA- and ECSA-based FACRM under gradations A and B can be systematically analyzed. Under gradation A, when the asphalt content is 3.5%, both the dry and wet indirect tensile strengths of CNOOA and ECSA reach their peak values. The dry and wet indirect tensile strengths of CNOOA are 0.65 MPa and 0.55 MPa, respectively, while those of ECSA are 0.52 MPa and 0.42 MPa, respectively. Notably, the indirect tensile strength ratios of the two mixtures also reach their highest levels at this asphalt content, with values of 84.6% and 80.8%, respectively, indicating that both asphalts exhibit good resistance to moisture damage at this content. For gradation B, the optimum asphalt content is 2.5%. Under this condition, the dry and wet indirect tensile strengths of CNOOA are 0.53 MPa and 0.42 MPa, respectively, while those of ECSA are 0.49 MPa and 0.38 MPa, respectively. The maximum indirect tensile strength ratios are 79.2% and 77.5%, respectively. Although the absolute strength values are lower than those under gradation A, the mixtures still exhibit acceptable moisture stability. Overall, at the same asphalt content, the dry and wet indirect tensile strengths of the mixtures prepared with CNOOA are significantly higher than those prepared with ECSA, with increases ranging from 8.2% to 26.1%. This indicates that CNOOA not only provides superior bonding performance but also enhances resistance to moisture damage. This performance difference is mainly attributed to the better foaming characteristics of CNOOA. Its longer half-life indicates that the foam can be maintained for a longer period, thereby extending the effective mixing and contact time with the recycled mixture and enabling more sufficient coating and more uniform dispersion. Meanwhile, the higher expansion ratio of CNOOA results in more pronounced volume expansion after foaming, greatly improving the distribution uniformity of foamed asphalt in the mixture. This allows CNOOA to more effectively coat fine aggregates and form a continuous and strong asphalt mortar, thereby significantly enhancing the overall bonding capacity and structural strength of the mixture. These findings are consistent with those reported in [10].

When the asphalt type is fixed, the dry and wet indirect tensile strengths, as well as the ITSR, initially increase and then decrease with increasing asphalt content [30]. This phenomenon is mainly attributed to the fact that an increase in asphalt content enables a more complete asphalt film to form on the aggregate surface, thereby enhancing the bonding between aggregates. When the asphalt content reaches the optimum value, such as 3.5% for gradation A and 2.5% for gradation B, the dry indirect tensile strength reaches its peak. At this point, the asphalt film has an appropriate thickness, which effectively bonds the aggregates without excessively weakening the interlocking effect among them. However, when the asphalt content continues to increase beyond the optimum range, the excessively thick asphalt film produces a lubricating effect, reducing the internal friction between aggregates and consequently leading to a decrease in dry indirect tensile strength.

Considering that the interval of asphalt content in the experiment is 0.5%, the recommended allowable range for the optimal asphalt content in engineering practice can be taken as ±0.25% around the peak value. Accordingly, for gradation A, the recommended optimum foamed asphalt content is 3.5%, with a suggested control range of 3.25–3.75%. For gradation B, the recommended optimum foamed asphalt content is 2.5%, with a suggested control range of 2.25–2.75%. Meanwhile, in practical engineering applications, these values should be further verified by considering on-site material variability, mixing uniformity, and the results of indirect tensile strength tests [51].

#### 3.2.2. Influence of Curing Conditions on Mixture Performance

**Figure 12:**
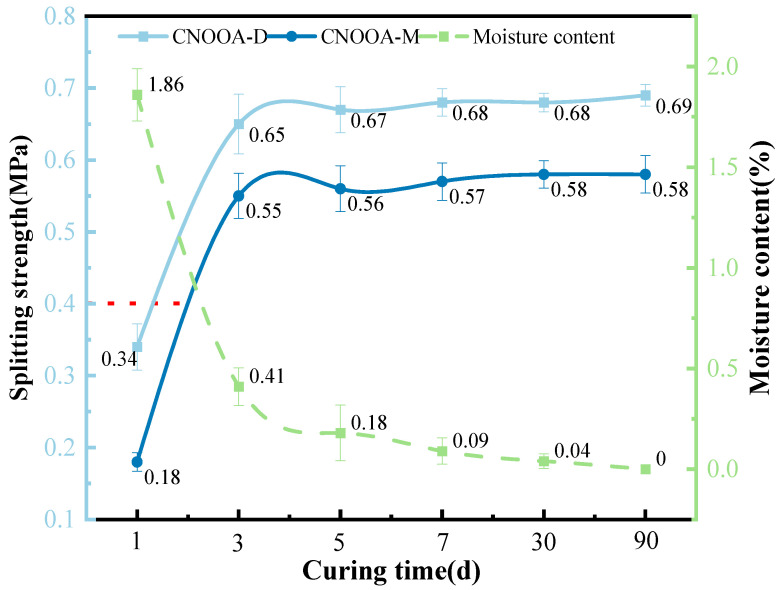
Relationship Between Curing Time, Indirect Tensile Strength and Moisture Content.

Figure 12 shows the test results of dry and wet indirect tensile strength and water content of FACRM under different curing times.

As shown in Figure 12, with increasing curing time, the moisture content of the mixture decreases rapidly, while the dry and wet indirect tensile strengths increase significantly. When the curing time is 1 day, the moisture content reaches the highest value of 1.86%, and the specimens exhibit certain strength levels, with dry and wet indirect tensile strengths of 0.34 MPa and 0.18 MPa, respectively. Compared with the maximum tensile strengths, these values are reduced by 50.72% and 68.97%, respectively. As the curing time increases from 1 day to 3 days, the dry and wet indirect tensile strengths show substantial growth, reaching 0.65 MPa and 0.55 MPa, respectively, corresponding to increases of 91.18% and 205.56%. Meanwhile, the moisture content decreases to 0.41%. When the curing time exceeds 3 days, the dry and wet indirect tensile strengths remain relatively stable, and both the tensile strength and moisture content show little variation with further curing time. This phenomenon indicates that the timely dissipation of moisture creates favorable conditions for strength development [17]. As excess free water in the mixture is removed, the asphalt film formed after the collapse of foamed asphalt can fully contact the aggregate particles, effectively enhancing its bonding effect. This promotes the transition of the mixture from an initial plastic state to an elastic state, while its internal structure becomes increasingly dense and strengthened. However, once the moisture content decreases to a certain threshold, the contribution of extended curing time to strength improvement becomes significantly limited, and the strength curve tends to level off. This critical behavior suggests the existence of an optimal curing period for strength development in the mixture. Excessive curing not only provides limited additional performance benefits but may also increase construction costs and time, which has important implications for optimizing curing regimes in engineering practice [52].

#### 3.2.3. Influence of Cement Content on Mixture Performance

**Figure 13:**
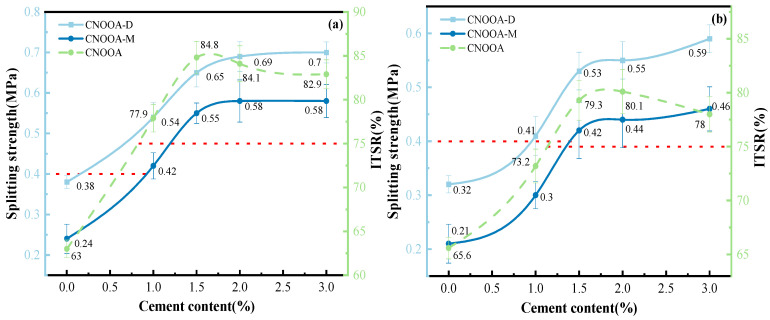
Change in Indirect Tensile Strength Under Different Gradations and Cement Contents: (**a**) Gradation A; (**b**) Gradation B.

Figure 13 shows the test results of the dry and wet indirect tensile strength of FACRM under different cement contents.

As shown in Figure 13, when the cement content increases from 0% to 1.5%, the dry and wet indirect tensile strengths and ITSR of FACRM gradually increase, with significant growth rates. For gradation A, the dry and wet indirect tensile strengths and ITSR increase from 0.38 MPa, 0.24 MPa, and 63% to 0.65 MPa, 0.55 MPa, and 84.8%, respectively, with maximum increases of 71.05%, 129.17%, and 34.60%. For gradation B, the corresponding values increase from 0.32 MPa, 0.21 MPa, and 65.6% to 0.53 MPa, 0.42 MPa, and 79.3%, with maximum increases of 65.63%, 100.00%, and 20.88%, respectively. When the cement content exceeds 1.5%, the dry and wet indirect tensile strengths of CNOOA-based mixtures gradually stabilize, while the ITSR shows an overall decreasing trend. When the cement content reaches 2%, the maximum ITSR for gradation B is 80.1%. In summary, with increasing cement content, the dry and wet indirect tensile strengths of FACRM under both gradations A and B exhibit a significant upward trend. The addition of cement effectively fills internal voids in the mixture and enhances the bonding between aggregates, thereby markedly improving the mechanical properties of the material [44]. It is noteworthy that the ITSR shows a trend of first increasing and then decreasing with increasing cement content, indicating the existence of an optimal cement content range. Excessive cement addition may adversely affect the moisture resistance of the mixture. Based on a comprehensive evaluation of the balance between tensile strength and ITSR, the recommended cement content for recycled mixtures under both gradations A and B is within the range of 1.5–2.0%. This range allows for full utilization of the synergistic enhancement effect of cement and asphalt while avoiding the potential negative impact of excessive cement on moisture stability, thereby ensuring optimal overall performance of the recycled mixture [45].

#### 3.2.4. Influence of Mixing Water Content on Mixture Performance

**Figure 14:**
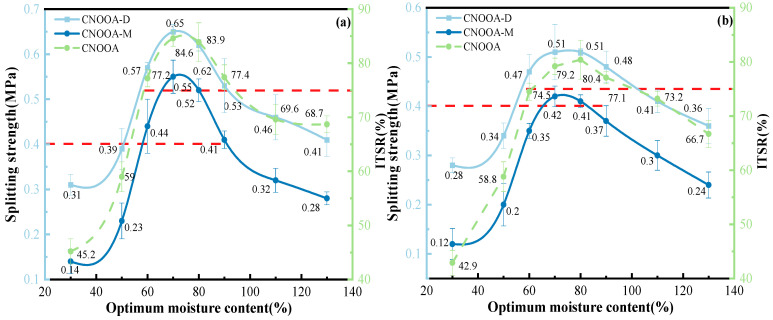
Indirect Tensile Strength Under Different Mixing Water Contents: (**a**) Gradation A; (**b**) Gradation B.

Figure 14 shows the relationship curves of dry indirect tensile strength, wet indirect tensile strength, and ITSR of FACRM under different mixing water contents.

As shown in Figure 14, when the mixing water content ranges from 30% to 70% of the OMC, the dry and wet indirect tensile strengths and ITSR increase significantly. For gradation A, the dry and wet indirect tensile strengths and ITSR increase from 0.31 MPa, 0.14 MPa, and 45.2% to 0.65 MPa, 0.55 MPa, and 84.6%, respectively, with maximum increases of 38.2%, 292.86%, and 37.3%. For gradation B, the corresponding values increase from 0.28 MPa, 0.12 MPa, and 42.9% to 0.51 MPa, 0.42 MPa, and 79.2%, respectively, with maximum increases of 46.2%, 250%, and 30.9%. When the mixing water content exceeds 70%, the dry and wet indirect tensile strengths and ITSR generally show a decreasing trend. For gradation A, when the mixing water content exceeds 90%, the wet indirect tensile strength falls below 0.4 MPa. Similarly, for gradation B, when the mixing water content exceeds 80%, the wet indirect tensile strength also drops below 0.4 MPa. Overall, the dry and wet indirect tensile strengths and ITSR of both gradations A and B exhibit a trend of initially increasing and then decreasing, indicating the existence of an optimal mixing water content for both gradations [46]. The optimal mixing water content ranges are 60–90% of the OMC for gradation A and 70–80% for gradation B. Within these ranges, the mixing performance is favorable, asphalt dispersion is uniform without agglomeration, compaction is effective, and the recycled mixtures exhibit high strength. Under different mixing water contents, both gradations show similar trends in dry and wet indirect tensile results, mainly because asphalt is well dispersed and compaction is effective within the optimal range. Mechanistically, insufficient water content leads to uneven asphalt dispersion and agglomeration, adversely affecting compaction [50]. Conversely, excessive water content may cause water bleeding, increase air voids, and thereby reduce both the strength and moisture stability of the mixture. Therefore, in practical engineering applications, the mixing water content should be carefully controlled according to the gradation type to optimize mixture performance.

### 3.3. Significance Analysis of Influencing Factors

As shown in Table 7, the correlation coefficients between indirect tensile strength and each variable are all greater than 0.4, indicating that indirect tensile strength is positively correlated with asphalt content, curing time, and cement content.

Multiple linear regression was further adopted to establish a prediction model for indirect tensile strength, aiming to quantify the degree of influence of each factor and reveal their mathematical relationships, thus providing theoretical support for engineering design and material ratio optimization. The regression analysis results are shown in Table 8, where T represents the T-statistic for each regression coefficient, and B represents the unstandardized partial regression coefficient of each variable. The p-values for asphalt content, curing time, and cement content are all less than 0.05, indicating that these three factors have statistically significant effects on indirect tensile strength. According to the standardized coefficients (0.315, 0.643, and 0.590, respectively), the order of influence degree of each factor on indirect tensile strength is: curing time > cement content > asphalt content.

(1) σ=−0.092+0.091∗A+0.067∗T+0.138∗C

The obtained multiple linear regression prediction model is shown in Equation (1), where *A* represents asphalt content, *T* represents curing time, and *C* represents cement content. The coefficient of determination (R^2^) of the model is 0.913, indicating that 91.3% of the variation in indirect tensile strength can be jointly explained by these three independent variables.

To verify the applicability of the model, diagnostic tests of model assumptions were conducted. In terms of sample independence, random sampling and grouped control were adopted during the experimental design stage to ensure the independence of data sources. The Durbin–Watson test yielded a statistic of 1.810. Based on the sample size (n = 16) and the number of predictor variables (k = 3), this value falls within the range indicating no autocorrelation (1.5–2.5), suggesting that there is no significant autocorrelation in the model residuals. In the multicollinearity diagnosis, the variance inflation factors (VIF) for all variables were less than 5, indicating that there is no severe multicollinearity among the independent variables.

It should be noted that the formula established in this study is mainly applicable to the preliminary estimation of indirect tensile strength under similar test conditions. Since it adopts standardized numerical modeling, it is convenient for horizontal comparison with relevant studies, but it still needs to be used cautiously in combination with actual engineering conditions.

## 4. Conclusions

(1) Asphalt type and foaming process are the key factors affecting foaming performance. Among the three types of 70# asphalt, CNOOA exhibits the best foaming performance. Under the optimal foaming condition of 160 °C and 2% foaming water content, it produces high-quality foam with an expansion ratio of 27 and a half-life of 30.3 s, which is significantly superior to KA and ECSA.
(2) Asphalt type and dosage are the core factors determining the indirect tensile strength of foamed asphalt cold recycled (FACRM) mixtures. Specifically, under the same gradation and dosage, the dry and wet indirect tensile strengths of mixtures prepared with CNOOA (which has better foaming characteristics) are 8.2% to 26.1% higher than those with Donghai asphalt. For both gradations A and B, the indirect tensile strength shows a trend of first increasing and then decreasing with the increase in asphalt content, peaking at 3.5% and 2.5% dosage, respectively.
(3) Curing time dominates the strength formation process of recycled mixtures by regulating water loss. The water content of the mixture decreases rapidly from 1.86% to 0.41% during the first 3 days of curing, while the dry and wet indirect tensile strengths increase significantly by 91.18% and 205.56%, respectively. After curing for more than 3 days, both the strength and water content tend to stabilize, indicating that 3 days is the critical period for strength development.
(4) Cement content and mixing water content have clear optimal ranges in terms of their effects on mixture performance, rather than increasing monotonically with dosage. When the cement content increases from 0% to 1.5%, the indirect tensile strength improves significantly; however, when the cement content exceeds 1.5%, the strength growth becomes limited, and the residual strength ratio begins to decrease. The mixture exhibits optimal performance when the mixing water content is approximately 70% of the OMC.
(5) There are significant differences in the degree of influence of various factors on the indirect tensile strength of the mixture. Based on multiple linear regression analysis, the standardized coefficients of curing time, cement content, and asphalt content are 0.643, 0.590, and 0.315, respectively, indicating the order of importance of their influence on indirect tensile strength is: curing time > cement content > asphalt content.

## Figures and Tables

**Table 1:** Test Results of Main Properties of Asphalt.

Technical Indicators	KA	CNOOA	ECSA	Technical Requirements	Test Method
25 °C Needle penetration (0.1mm)	68	64	72	60~80	T0604 [32]
Penetration Index (PI)	−0.94	−1.15	−0.85	−1.5~+1.0	T0604 [32]
15 °C Extension(cm)	>150	>150	>150	≥100	T0605 [31]
Softening point (℃)	48.1	47.7	47.5	≥46	T0606 [31]
15 °C Density (g/cm^3^)	1.026	1.012	1.034	Measured	T0603 [31]
60 °C dynamic viscosity (Pa·s)	238	236	240	≥180	T0620 [31]
Mass loss after RTFOT	−0.19	−0.16	−0.18	≤±0.8	T0609 [31]
Flash point (℃)	278	274	285	≥260	T0611 [31]
Solubility (%)	99.91	99.90	99.93	≥99.5	T0607 [31]

**Table 2:** Proportions of Combined Aggregate Gradation of Recycled Mixture.

Gradation Type	Mass Percentage of Each Grade of Aggregate (mm) in the Total Aggregate/%			
	Mineral Filler	Crushed Stone Dust (0~3 mm)	Reclaimed Aggregate (10~30 mm)	Reclaimed Aggregate (0~10 mm)
Gradation A	20	20	45	30
Gradation B	0	20	45	35

**Table 3:** Main Technical Indicators of Cement.

Test Item	Unit	Standard Requirement	Test Result	Test Method
Fineness retained on 80 μm square-hole sieve	%	≤10.0	12	GB 8074 [34]
Initial setting time	min	≥45	110	GB1346 [35]
Final setting time	h	≤10	4.3	
Soundness, boiling method	—	Qualified	Qualified	
3 d compressive strength	MPa	≥32.5	20.6	JC/T683 [36]
28 d compressive strength		≥2.5	46.1	
3 d flexural strength		5.5	53	JC/T724 [37]
28 d flexural strength		≤10.0	8.7	

**Table 4:** Compaction Test Results and Mixing Water Content.

Gradation Type	Optimum Moisture Content (%)	Maximum Dry Density (g/cm^3^)	Optimum Mixing Water Content (%)	Optimum Foamed Asphalt Content (%)
Gradation A	6.32	2.213	4.42	3.5
Gradation B	5.60	2.186	3.92	2.5

**Table 5:** Specific Parameter Information.

Asphalt Type	Temperature (℃)			Foaming Water Content (%)			
KA	150	160	170	1	2	3	4
CNOOA	150	160	170	1	2	3	4
ECSA	150	160	170	1	2	3	4

**Table 6:** Main Parameters and Their Value Ranges.

Gradation Type	CNOOA								
Gradation A	Asphalt content (%)	2.0	2.5	3.0	3.5	4			
	Mixing water content (%)	30	50	60	70	80	90	110	130
	Curing time (d)	1	3	5	7	30	90		
	Cement content (%)	0	1	1.5	2	3			
Gradation B	Asphalt content (%)	1.0	1.5	2.0	2.5	3.0			
	Mixing water content (%)	30	50	60	70	80	90	110	130
	Curing time (d)	1	3	5	7	30	90		
	Cement content (%)	0	1	1.5	2	3			

**Table 7:** Pearson Correlation Coefficients.

	Indirect Tensile Strength	Asphalt Content	Curing Time	Cement Content	Sample Size
Indirect tensile strength	1.000				-
Asphalt content	0.428	1.000			6
Curing time	0.699	0.176	1.000		5
Cement content	0.590	0.000	0.000	1.000	5

**Table 8:** Multiple Linear Regression Analysis.

Parameter	Unstandardized Coefficient		Standardized Coefficient Beta	T	Significance	Collinearity Statistics	
	B	Standard Error				Tolerance	VIF
Constant	−0.092	0.085		−1.091	0.301		
Asphalt content	0.091	0.024	0.315	3.791	0.004	0.969	1.032
Curing time	0.067	0.009	0.643	7.735	0.000	0.969	1.032
Cement content	0.138	0.019	0.590	7.211	0.000	1.000	1.000

## Data Availability

The original contributions presented in this study are included in the article. Further inquiries can be directed to the corresponding author.
